# Recombinant methioninase (rMETase) is an effective therapeutic for BRAF-V600E-negative as well as -positive melanoma in patient-derived orthotopic xenograft (PDOX) mouse models

**DOI:** 10.18632/oncotarget.23185

**Published:** 2017-12-12

**Authors:** Kei Kawaguchi, Kentaro Igarashi, Shukuan Li, Qinghong Han, Yuying Tan, Kentaro Miyake, Tasuku Kiyuna, Masuyo Miyake, Takashi Murakami, Bartosz Chmielowski, Scott D. Nelson, Tara A. Russell, Sarah M. Dry, Yunfeng Li, Michiaki Unno, Fritz C. Eilber, Robert M. Hoffman

**Affiliations:** ^1^ AntiCancer, Inc., San Diego, CA, USA; ^2^ Department of Surgery, University of California, San Diego, CA, USA; ^3^ Department of Surgery, Graduate School of Medicine, Tohoku University, Sendai, Japan; ^4^ Division of Hematology-Oncology, University of California, Los Angeles, CA, USA; ^5^ Department of Pathology, University of California, Los Angeles, CA, USA; ^6^ Division of Surgical Oncology, University of California, Los Angeles, CA, USA

**Keywords:** melanoma, recombinant methioninase, methionine dependence, BRAF-V600E mutation, PDOX

## Abstract

Melanoma is a recalcitrant disease. Melanoma patients with the BRAF-V600E mutation have been treated with the drug vemurafenib (VEM) which targets this mutation. However, we previously showed that VEM is not very effective against a BRAF-V600E melanoma mutant in a patient-derived orthotopic xenograft (PDOX) model. In contrast, we demonstrated that recombinant methioninase (rMETase) which targets the general metabolic defect in cancer of methionine dependence, was effective against the BRAF-V600E mutant melanoma PDOX model. In the present study, we demonstrate that rMETase is effective against a BRAF-V600E-negative melanoma PDOX which we established. Forty BRAF-V600E-negative melanoma PDOX mouse models were randomized into four groups of 10 mice each: untreated control (*n* = 10); temozolomide (TEM) (25 mg/kg, p.o., 14 consecutive days, *n* = 10); rMETase (100 units, i.p., 14 consecutive days, *n* = 10); TEM + rMETase (TEM: 25 mg/kg, p.o., rMETase: 100 units, i.p., 14 consecutive days, *n* = 10). All treatments inhibited tumor growth compared to untreated control (TEM: *p* = 0.0003, rMETase: *p* = 0.0006, TEM/rMETase: *p* = 0.0002) on day 14 after initiation. Combination therapy of TEM and rMETase was significantly more effective than either mono-therapy (TEM: *p* = 0.0113, rMETase: *p* = 0.0173). The present study shows that TEM combined with rMETase is effective for BRAF-V600E-negative melanoma PDOX similar to the BRAF-V600E-positive mutation melanoma. These results suggest rMETase in combination with first-line chemotherapy can be highly effective in both BRAF-V600E-negative as well as BRAF-V600E-positive melanoma and has clinical potential for this recalcitrant disease.

## INTRODUCTION

Melanoma is a recalcitrant cancer [1] with no cure for stage III and IV due to many factors including drug resistance, tumor heterogeneity and an immune-suppressed tumor microenvironment [2].

Temozolomide (TEM), an alkylating agent, is first-line chemotherapy for melanoma but with limited efficacy [1–5].

An excessive requirement for methionine (MET) termed MET dependence, appears to be a general metabolic defect in cancer. MET restriction therapy, using recombinant methioninase (rMETase), sensitized brain tumors to TEM in xenografts in nude mice [6]. In a previous study, we tested a patient-derived orthotopic xenograft (PDOX) nude mouse model of BRAF V600E-mutant melanoma and observed the efficacy of rMETase [7].

In the present study, we established a PDOX nude mouse model with a BRAF-V600E-negative melanoma from a patient. We evaluated the efficacy of rMETase and rMETase in combination with TEM on the BRAF-V600E-negative melanoma.

## RESULTS AND DISCUSSION

**Figure 1:**
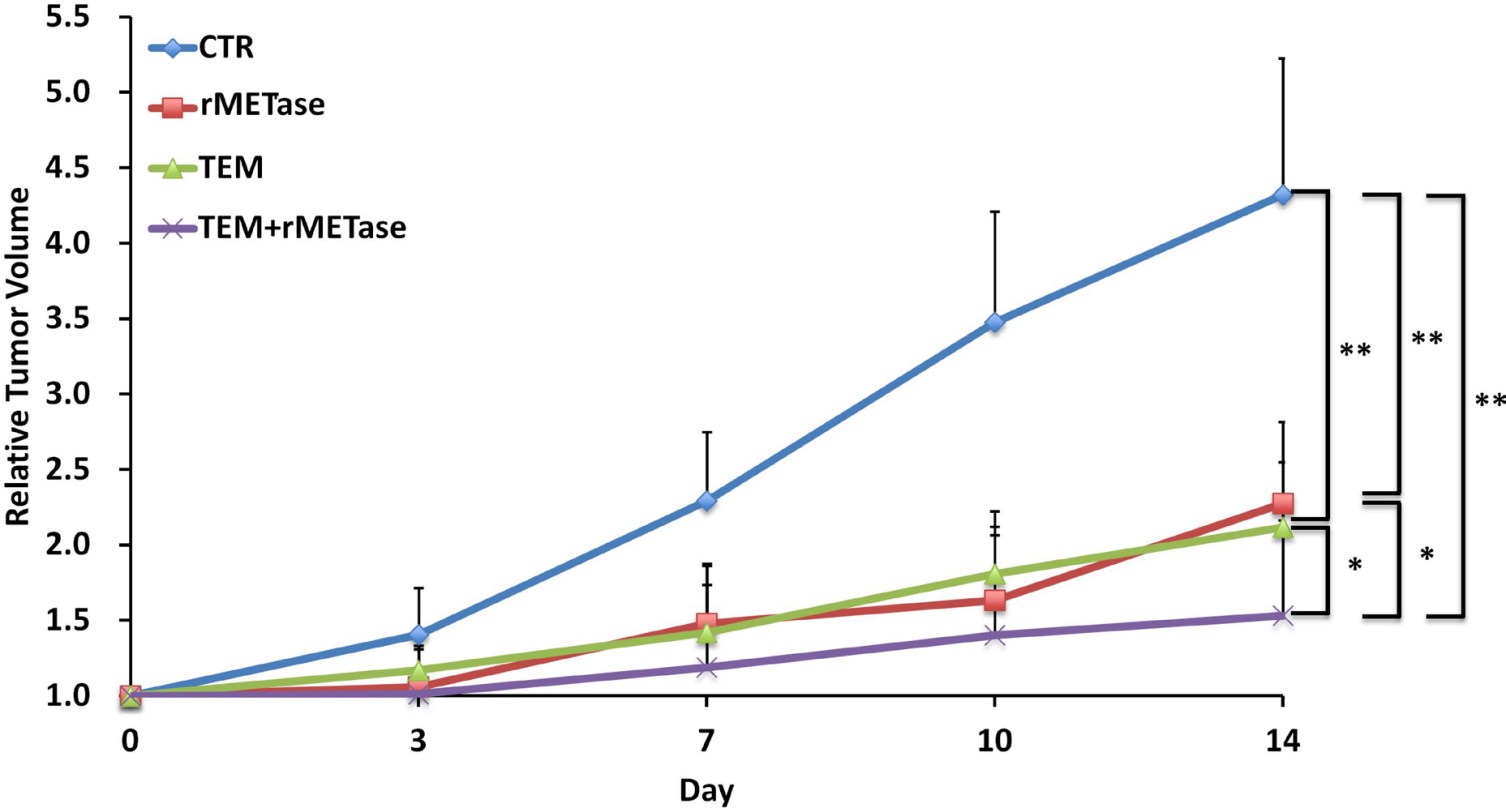
Quantitative efficacy of chemotherapy on the BRAF-V600E-negative PDOX Line graphs show relative tumor volume at each point relative to the initial tumor volume for each treatment and control group. ^*^p < 0.02; ^**^p < 0.01. Error bars: ± SD.

Temozolomide (TEM) (*p =* 0.0003), rMETase (*p =* 0.0006) and the combination of TEM/rMETase (*p =* 0.0002) inhibited tumor growth in the BRAF-V600E-negative PDOX, as measured on day 14 after initiation. The combination of TEM and rMETase was significantly more effective than TEM (*p =* 0.0113) and rMETase (*p =* 0.0173) alone. There was no significant difference between TEM and rMETase alone (*p* = 0.4274) (Figure 1).

**Figure 2:**
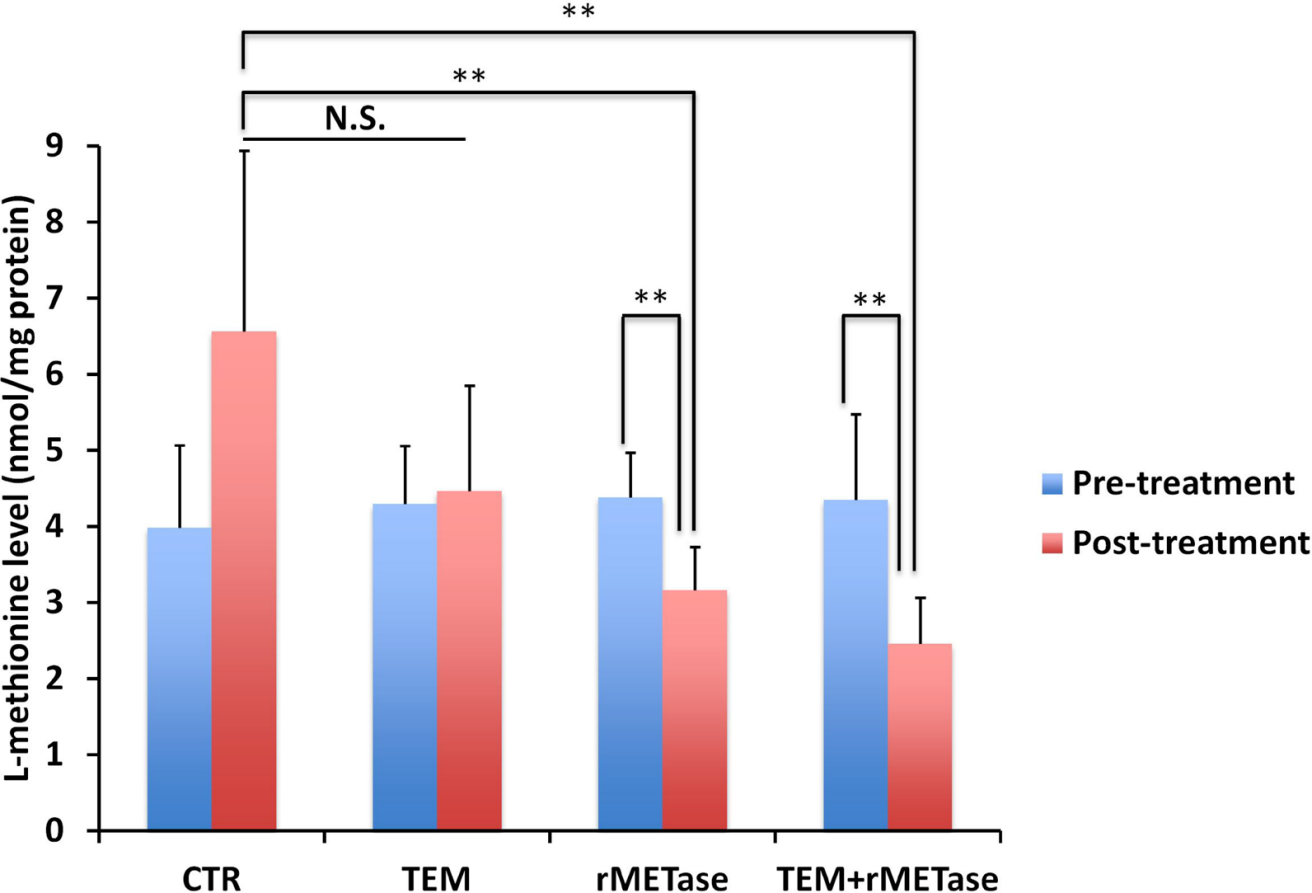
Intra-tumor MET levels Bar graphs show MET level in each treatment or control group at pre- and post-treatment time points. ^**^p < 0.01.

Post-treatment MET levels in tumors treated with rMETase alone (*p =* 0.0009) or in combination with TEM (*p =* 0.0006) were significantly decreased compared to the untreated control (Figure 2), suggesting the lowering of tumor MET levels by rMETase is the mechanism of inhibition.

These results showed that this BRAF-V600E-negative melanoma PDOX is MET dependent and rMETase suppresses its growth, especially in combination with first-line chemotherapy TEM.

**Figure 3:**
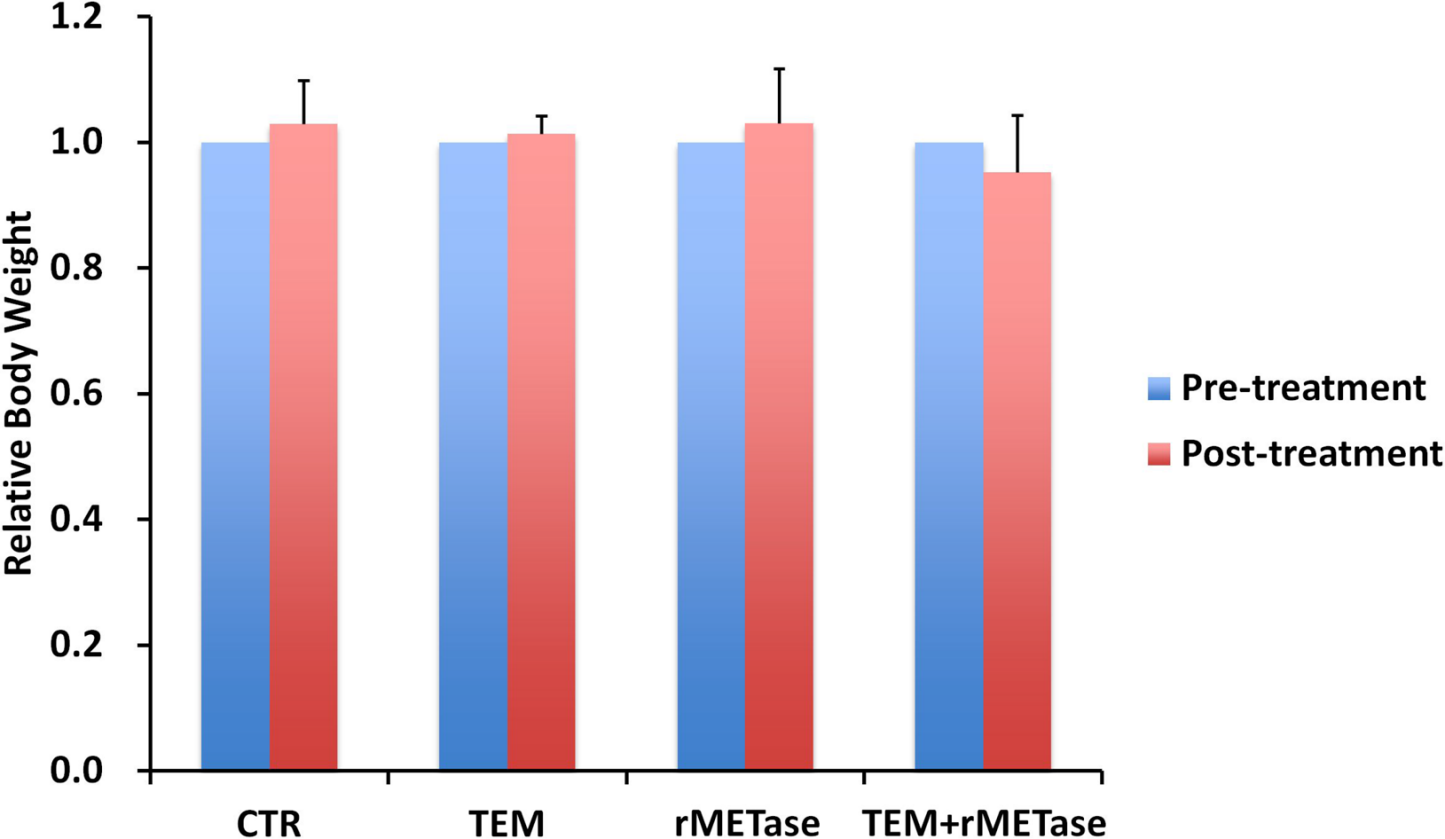
Mouse body weight Bar graphs show mouse body weight in each treatment or control group at pre- and post-treatment time points.

Slight body weight loss was observed only in the combination treatment group, however there were no significant difference in body weight between any group (Figure 3). There were no animal deaths in any group.

**Figure 4:**
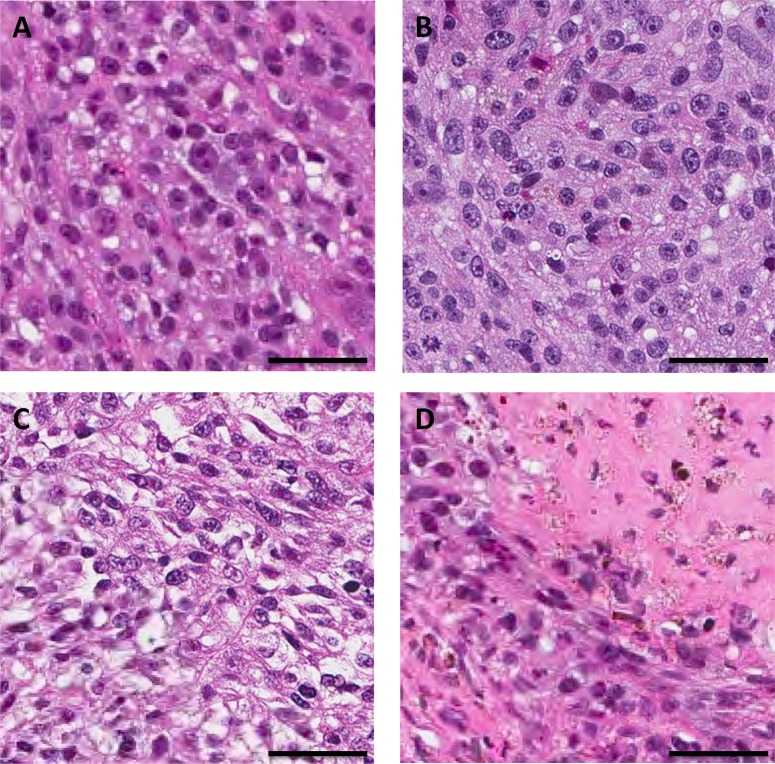
Tumor histology (**A**) Untreated control. (**B**) TEM treated. (**C**) rMETase treated. (**D**) TEM/rMETase treated. Scale bars: 50 μm.

Histologically, the untreated control tumor was mainly comprised of viable cells. The viable cancer cells of the untreated control specimen and the treated specimens had a similar appearance. The cancer cells display epithelioid to slightly spindly morphology, and contain moderate amounts of eosinophilic cytoplasm, with some cytoplasmic vacuolization present. Nuclei are moderately pleomorphic with 1 to 2 prominent nucleoli. Mitotic activity was present. The same slight degree of necrosis was observed in tumors treated with TEM and rMETase. In contrast, tumors treated with the combination of TEM and rMETase showed extensive necrosis (Figure 4).

TEM has been widely used as standard chemotherapy for BRAF-V600E-negative melanoma [1–5]. In this study, TEM inhibited tumor growth to a certain extent. TEM in combination with rMETase was significantly more effective than TEM alone, similar to our previous study for VEM resistant BRAF-V600E-positive melanoma [7].

The present study has important implications since this is the first report that TEM combined with rMETase is effective treatment for melanoma regardless of BRAF-V600E mutation status.

Effective individualized therapy requires matching the cancer patient with an effective drug. Toward this goal, our laboratory pioneered the patient-derived orthotopic xenograft (PDOX) nude mouse model with the technique of surgical orthotopic implantation (SOI), including breast [8], ovarian [9], lung [10], cervical [11, 12], colon [13–15], stomach [16], pancreatic [17–21], melanoma [22–26], and sarcoma [27–36]. The PDOX model, developed by our laboratory over the past 30 years, has many advantages over subcutaneous-transplant models which are growing ectopically under the skin [37]. The PDOX model enables precise, individualized therapy, especially for recalcitrant diseases such as melanoma [23] by matching the patient tumor to an effective drug identified with the PDOX models.

Methionine dependence is due to excess use of methionine for aberrant transmethylation reactions [38, 39]. The elevated methionine use in cancer cells has been termed the “Hoffman effect” [29, 39] analogous to the “Warburg effect” [40] for elevated aerobic glucose use in cancer. The excessive and aberrant use of methionine in cancer is observed in [^11^C]methionine PET imaging, where high uptake of [^11^C]methionine results in a very strong and selective tumor signal compared to normal tissue background for brain and other cancers [39]. In a comparison of MET-PET and fluorodeoxyglucose (FDG)-PET, MET-PET was found to be superior for glioma [41–46] suggesting that cancer may have a greater abnormal requirement for methionine than glucose [39].

Cell lines derived from various cancer types including liver, pancreatic ovarian, submaxillary, brain, lung, bladder, prostate, breast, kidney, cervical, colon, fibrosarcoma, osteosarcoma, rhabdomyosarcoma, leiomyosarcoma, neuroblastoma, glioblastoma and melanoma were shown to be methionine dependent in contrast to normal cell lines. Human patient tumors, including tumors of the colon, breast, ovary, prostate, and a melanoma, were also found to be methionine dependent in Gelfoam^®^ histoculture [39, 47].

We reported recently on efficacy of rMETase against Ewing’s sarcoma in a PDOX model. rMETase effectively reduced tumor growth compared to untreated control [29]. We also recently reported that TEM combined with rMETase could strongly inhibit tumor growth of BRAF-V600E-positive melanoma PDOX [29]. The occurrence of methionine dependence among many diverse cancer types suggests that methionine dependence is a general phenomena in cancer [39].

Previously-developed concepts and strategies of highly-selective tumor targeting can take advantage of molecular targeting of tumors, including tissue-selective therapy which focuses on unique differences between normal and tumor tissues [48–53].

## CONCLUSIONS

The present study has demonstrated the efficacy of rMETase and the combination of rMETase and TEM on a BRAF-V600E-negative melanoma PDOX model. These results sugest that both BRAF-V600E-negative as well as -positive melanoma are sensitive to rMETase. Further PDOX experiments will determine the general efficacy of rMETase on melanoma as a prelude to its clinical development for this recalcitrant disease.

Both classical and novel forms of vitamin D have shown efficacy against melanoma progression and management, including in the adjuvant setting [54, 55]. Future experiments will evaluate rMETase along with classical and novel forms of vitamin D. We recently reviewed the establishment and properties of PDOX models of melanoma [56].

## MATERIALS AND METHODS

### Mice

Athymic *nu/nu* nude mice (AntiCancer Inc., San Diego, CA), 4–6 weeks old, were used in this study. Mice were housed in a barrier facility on a high efficacy particulate arrestance (HEPA)-filtered rack under standard conditions of 12-hour light/dark cycles. The animals were fed an autoclaved laboratory rodent diet. All animal studies were conducted in accordance with the principles and procedures outlined in the National Institutes of Health Guide for the Care and Use of Animals under Assurance Number A3873-1. All mouse surgical procedures and imaging were performed with the animals anesthetized by subcutaneous injection of a ketamine mixture (0.02 ml solution of 20 mg/kg ketamine, 15.2 mg/kg xylazine, and 0.48 mg/kg acepromazine maleate). The response of animals during surgery was monitored to ensure adequate depth of anesthesia. The animals were observed on a daily basis and humanely sacrificed by CO_2_ inhalation if they met the following humane endpoint criteria: severe tumor burden (more than 20 mm in diameter), prostration, significant body weight loss, difficulty breathing, rotational motion and body temperature drop [7].

### Patient-derived tumor

A patient diagnosed with a BRAF-600E-negative melanoma of the abdominal wall was resected in the Department of Surgery, University of California, Los Angels (UCLA). Written informed consent was provided by the patient, and the Institutional Review Board (IRB#10-001857) of UCLA approved that the patient’s resected melanoma could be used for the present study. The tumor was designated at PDOX063.

### Establishment of PDOX models of melanoma by surgical orthotopic implantation (SOI)

A fresh sample of the melanoma of the patient was obtained and transported immediately to the laboratory at AntiCancer, Inc., on wet ice. The sample was cut into 5-mm fragments and implanted subcutaneously in nude mice. After three weeks, the subcutaneously-implanted tumors grew to more than 10 mm in diameter. The subcutaneously-grown tumors were then harvested and cut into small fragments (3 mm^3^). After nude mice were anesthetized with the ketamine solution described above, a 5-mm skin incision was made on the abdominal wall, which was split to make space for the melanoma tissue fragment. A single tumor fragment was implanted orthotopically into the space to establish the PDOX model. The wound was closed with a 6-0 nylon suture (Ethilon, Ethicon, Inc., NJ, USA) [23, 24].

### Recombinant methionase (rMETase) production

Recombinant L-metionine α-deamino-γ-mercapto-methane lyase (recombinant methioninase, [rMETase]) [EC 4.4.1.11] from *Pseudomonas putida* has been previously cloned and was produced in *Escherichia coli* (AntiCancer, Inc., San Diego, CA). rMETase is a homotetrameric PLP enzyme of 172-kDa molecular mass [57].

### Treatment study design in the PDOX model of melanoma

PDOX mouse models of BRAF-V600E-negative melanoma were randomized into four groups of 10 mice each: untreated control (*n* = 10); TEM (25 mg/kg, oral [p.o.], 14 consecutive days, *n* = 10); rMETase (100 units, intraperitoneal [i.p.], 14 consecutive days, *n* = 10); TEM + rMETase (TEM: 25 mg/kg, p.o., rMETase: 100 units, i.p., 14 consecutive days, *n* = 10). Tumor length and width were measured twice a week. Tumor volume was calculated with the following formula: Tumor volume (mm^3^) = length (mm) × width (mm) × width (mm) × 1/2. Data are presented as mean ± SD. The tumor volume ratio is defined at the tumor volume at each point relative to pre-treatment tumor volume.

### Intra-tumor MET level analysis

Each tumor, untreated or treated, was sonicated for 30 seconds on ice and centrifuged at 12,000 rpm for 10 minutes. Supernatants were collected and protein levels were measured using the Coomassie Protein Assay Kit (Thermo Scientific, Rockford, IL). Protein levels were calculated from the standard curve obtained by protein standard, bovine serum albumin (BSA). MET levels were determined with an HPLC procedure described previously [29, 58]. Standardized MET levels were calculated using the following formula: MET level (nmol/mg protein) = MET level (nmol/ml) / protein level (mg protein/ml) [7].

### Histological examination

Fresh tumor samples, both untreated and treated, were fixed in 10% formalin and embedded in paraffin before sectioning and staining. Tissue sections (5 μm) were deparaffinized in xylene and rehydrated in an ethanol series. Hematoxylin and eosin (H&E) staining was performed according to standard protocols. Histological examination was performed with a BHS System Microscope (Olympus Corporation, Tokyo, Japan). Images were acquired with INFINITY ANALYZE software (Lumenera Corporation, Ottawa, Canada) [23–26].

### Statistical analysis

JMP version 11.0 was used for all statistical analyses. Significant differences for continuous variables were determined using the Mann-Whitney *U* test. Line graphs express average values and error bars show SD. A probability value of *P* ≤ 0.05 was considered statistically significant [7].
